# Retraction: MicroRNA-379 inhibits the proliferation, migration and invasion of human osteosarcoma cells by targetting EIF4G2

**DOI:** 10.1042/BSR-20160542_RET

**Published:** 2021-04-16

**Keywords:** EIF4G2, Invasion, MicroRNA-379, Migration, Osteosarcoma, Proliferation

This Retraction follows an Expression of Concern and Correction relating to this article previously published by Portland Press.

This article is being retracted from *Bioscience Reports* at the request of the Editor-in-Chief and the Editorial Board following receipt of a notification from several readers, alerting the Editorial Board to multiple image duplications in the original versions of Figures 5B, 5D, 5F and 5H.

The authors were contacted regarding the concerns raised and provided new replacement Figures; however, given that there were extensive duplications within the original and corrected images, the Editorial Board stands by the decision to retract the article. The authors agree to the retraction.

